# Prognostic importance and determinants of uremic pruritus in patients receiving peritoneal dialysis: A prospective cohort study

**DOI:** 10.1371/journal.pone.0203474

**Published:** 2018-09-05

**Authors:** Hon-Yen Wu, Jenq-Wen Huang, Wan-Chuan Tsai, Yu-Sen Peng, Hung-Yuan Chen, Ju-Yeh Yang, Shih-Ping Hsu, Mei-Fen Pai, Mei-Ju Ko, Kuan-Yu Hung, Hsien-Ching Chiu

**Affiliations:** 1 Department of Internal Medicine, Far Eastern Memorial Hospital, New Taipei City, Taiwan; 2 Institute of Epidemiology and Preventive Medicine, National Taiwan University, Taipei City, Taiwan; 3 Faculty of Medicine, School of Medicine, National Yang-Ming University, Taipei City, Taiwan; 4 Department of Internal Medicine, National Taiwan University Hospital and College of Medicine, Taipei City, Taiwan; 5 Department of Marketing and Distribution Management, Oriental Institute of Technology, New Taipei City, Taiwan; 6 Department of Dermatology, National Taiwan University Hospital and College of Medicine, Taipei City, Taiwan; 7 Department of Dermatology, Taipei City Hospital, Taipei City, Taiwan; 8 Department of Internal Medicine, National Taiwan University Hospital Hsin-Chu Branch, Hsinchu City, Taiwan; National Yang-Ming University, TAIWAN

## Abstract

**Background:**

Uremic pruritus is a common and frustrating symptom among patients receiving peritoneal dialysis (PD). This study aimed to examine the prognostic importance of uremic pruritus and to identify the determinants for higher pruritus intensity in PD patients.

**Methods:**

We conducted a prospective cohort study of patients receiving maintenance PD. A visual analogue scale (VAS) score was used to measure the intensity of uremic pruritus. The composite endpoint of PD technique failure or all-cause death was assessed using a multivariable Cox proportional hazards model. The determinants for the VAS score of uremic pruritus was assessed using a multivariable linear regression model.

**Results:**

Among the 85 PD patients, 24 (28%) had uremic pruritus. During a median follow-up of 28.0 months, 12 patients experienced technique failure, and 7 died. We found that a higher VAS score of pruritus intensity was an independent risk factor for technique failure or death (hazard ratio, 1.64; 95% confidence interval, 1.18 to 2.28; *P* = 0.003) after adjusting for a variety of confounding factors. We also found that a weekly total Kt/V of less than 1.88, a longer duration of dialysis, a higher dietary protein intake, and higher blood levels of intact parathyroid hormone and high-sensitivity C-reactive protein were independent determinants of higher VAS scores of pruritus intensity.

**Conclusions:**

Our results show that uremic pruritus is an independent risk factor of technique failure and death in patients receiving PD. We also found that a weekly total Kt/V < 1.88 is associated with higher intensity of uremic pruritus in PD patients.

## Introduction

Uremic pruritus is one of the most common and frustrating symptoms among patients with end-stage renal disease (ESRD), with prevalence ranging from 10% to 70% among patients receiving peritoneal dialysis (PD) [1,2]. Patients with pruritus suffer from sleep disturbance, depression, anxiety, uncontrollable itching, and impaired quality of life [3,4]. In patients receiving hemodialysis (HD), divalent ions, calcium-phosphate products, hyperparathyroidism, and interleukin-31 have been reported to be associated with uremic pruritus [5–9]. However, the pathophysiology of uremic pruritus remains obscure, and the therapeutic options are limited and unsatisfactory to PD patients.

Our previous study showed that the dialysis modality affects the prevalence and severity of uremic pruritus [10], suggesting that the pathogenesis of pruritus may differ between patients receiving PD and those on HD. Many predictive factors of uremic pruritus, such as age, comorbid diseases, nutritional status, solute clearance, and peritoneal transport properties, affect outcome in dialysis patients, and some factors are distinct in PD patients [11–16]. Although uremic pruritus is associated with mortality in patients receiving HD [9,17], the impact of uremic pruritus on technique failure and mortality in PD patients has never been investigated. Moreover, the relationships between uremic pruritus, biochemical parameters, and dialysis adequacy in patients receiving PD have rarely been reported.

Here, we report a prospective cohort study that examined the prognostic importance of uremic pruritus in patients receiving PD, and also identified the determinants for higher pruritus intensity in the cross-sectional part of the study.

## Materials and methods

### Study participants

We conducted a prospective cohort study of patients receiving maintenance PD at the Far Eastern Memorial Hospital, a tertiary medical center in Taiwan. There were 98 patients receiving maintenance PD. Patients were excluded if they were aged less than 20 years, had communication problems, were diagnosed with other primary skin disorders, or refused to participate in the study. We also excluded those with an active infection, an active malignancy, a psychotic illness, or a pregnancy. After excluding 12 patients who refused to participate and 1 patient with a psychotic illness, a total of 85 patients were enrolled and assessed in May 2013, and followed until August 2015.

### Ethics

This study protocol complied with the Declaration of Helsinki. The Institutional Review Board of the Far Eastern Memorial Hospital, New Taipei City, Taiwan, approved this prospective cohort study, and all participants provided written informed consent.

### Patient characteristics

Patient data, including age, sex, body mass index (BMI), comorbid diseases, etiology of ESRD, results of blood laboratory tests, PD assessments, and duration of dialysis therapy, were recorded for each participant. Systolic and diastolic blood pressure levels were measured during routine visits. Hypertension was defined as a systolic blood pressure ≥ 140 mmHg and/or a diastolic blood pressure ≥ 90 mmHg [18,19]. BMI was calculated using the following formula: [weight (kg) / height (m)^2^ ]. Venous blood was sampled after an overnight fast exceeding 8 hours. Patients with a positive test result for hepatitis B virus surface antigen were considered hepatitis B carriers, and those with a positive result for hepatitis C virus antibody were considered hepatitis C carriers. All laboratory tests were performed by the hospital’s central laboratory, and auto-analyzers were used to determine the biochemical data.

### Pruritus assessment

The patients were considered to have uremic pruritus if they had (1) at least three episodes of pruritus during a period of less than 2 weeks, with the symptoms occurring a few times a day, lasting at least a few minutes, and troubling the patient; or (2) the regular occurrence of pruritus during a period of 6 months but with a lower frequency than in (1) [2,20]. A visual analogue scale (VAS) score, which is a 10-cm line on which patients indicate the intensity of pruritus by marking the line at the point that corresponds to the severity of their pruritus, was reported from 0 to 10 (0 = no itching, 10 = worst imaginable itching) [21].

### Dialysis adequacy of solute clearance

The PD regimen and modality for each patient were evaluated and prescribed in the PD unit during monthly follow-ups. Dialysis solute clearance was assessed based on the Kt/V parameter (amount of dialysis delivered: K = clearance of urea, t = time on dialysis, V = estimated total body water) using urea kinetic modeling. The adequacy of solute clearance was assessed based on the weekly total Kt/V (the sum of peritoneal Kt/V and renal Kt/V) and should be at least 1.7 or above [22,23]. The transport properties of the peritoneal membrane were determined using the standard peritoneal equilibration test, which measures net fluid removal and the ratio of dialysate creatinine to blood creatinine after 4 hours [24]. Dietary protein intake was assessed based on protein nitrogen appearance and was normalized to the ideal body weight [25].

### Outcomes

This study had two aims. Firstly, a prospective cohort study design was used to examine the prognostic importance of VAS score of uremic pruritus. Secondly, a cross-sectional study design was used to explore the determinants associated with higher pruritus intensity based on the clinical and biochemical parameters of participants and their pruritus assessments at baseline. The status of the patients was recorded as permanent transfer to hemodialysis, death, renal transplantation, transfer to other hospitals, or no change in treatment status as of the last recorded follow-up date. Permanent transfer to hemodialysis was considered PD technique failure. The primary outcome was the composite endpoint of PD technique failure or all-cause death, and renal transplantation and hospital transfer were regarded as right-censoring events in the cohort study. The secondary outcome was the determinants for the VAS score of uremic pruritus using the cross-sectional baseline data.

### Statistical analysis

Statistical analysis was performed using R 2.14.1 software (R Foundation for Statistical Computing, Vienna, Austria). The data are expressed as the mean ± standard deviation for normally distributed continuous variables, as the median (1st and 3rd quartiles) for non-normally distributed continuous variables, and as numbers (percentages) for categorical variables. Continuous data were tested for normality by the Shapiro–Wilk test. For the descriptive analysis, univariable analyses were conducted using the independent two-sample *t*-test, the Wilcoxon rank-sum test, and Pearson’s chi-square test, respectively. The Kaplan-Meier method and the log-rank test were used to compare event-free survival between patients with and without pruritus. Multivariable Cox proportional hazards models were used to estimate the hazard ratio (HR) and 95% confidence interval (CI) of the VAS score of uremic pruritus for the composite endpoint of technique failure or all-cause death. Multivariable linear regression analysis was conducted to identify the determinants for the VAS score of uremic pruritus.

To find the parsimonious regression models that fitted the observed data well for effect estimation and ensure a good quality of analysis, basic model-fitting techniques for (1) variable selection, (2) goodness-of-fit assessment, and (3) regression diagnostics were used in the regression analyses. Specifically, the stepwise variable selection procedure (with iterations between the forward and backward steps) was applied to obtain the best candidate final regression model. All the significant and non-significant covariates were put on the variable list to be selected. The significance levels for entry (SLE) and for stay (SLS) were set to 0.15 for being conservative. Then, with the aid of clinical evidence, the best candidate final regression model was identified by dropping the covariates with *P* value > 0.05 one at a time until all regression coefficients were significantly different from 0. Since the statistical testing at each step of the stepwise variable selection procedure was conditioning on the other covariates in the regression model, the multiple testing problem was not of concern.

The generalized additive models (GAMs) were fitted to detect nonlinear effects of continuous covariates and identify appropriate cutoff points for discretizing continuous covariates, if necessary, during the stepwise variable selection procedure. Computationally, the vgam() function (with the default values of smoothing parameters) of the VGAM package [26–28] was used to fit GAMs for continuous, binary, and count responses in R. Since GAMs were originally developed for smoothing the effects of continuous covariates in generalized linear models (GLMs), we fitted GAMs of binary responses for survival outcomes. Alternatively, we specified the smoothing option pspline (for the smoothing splines using a “p-spline” basis) inside the coxph() function of the survival package to smooth the effects of continuous covariates on the survival outcome of Cox’s proportional hazards models in R [29]. The concordance and the *R*^*2*^ were examined to assess the goodness-of-fit of the Cox proportional hazards model and the linear regression model, respectively. Statistical tools for regression diagnosis, including verification of proportional hazards assumption, residual analysis, the detection of influential cases, and test for multicollinearity by checking variance inflation factors, were used to assess problems within the model or data. A two-sided *P* value ≤ 0.05 was considered statistically significant.

## Results

### Patient characteristics

**Fig 1:**
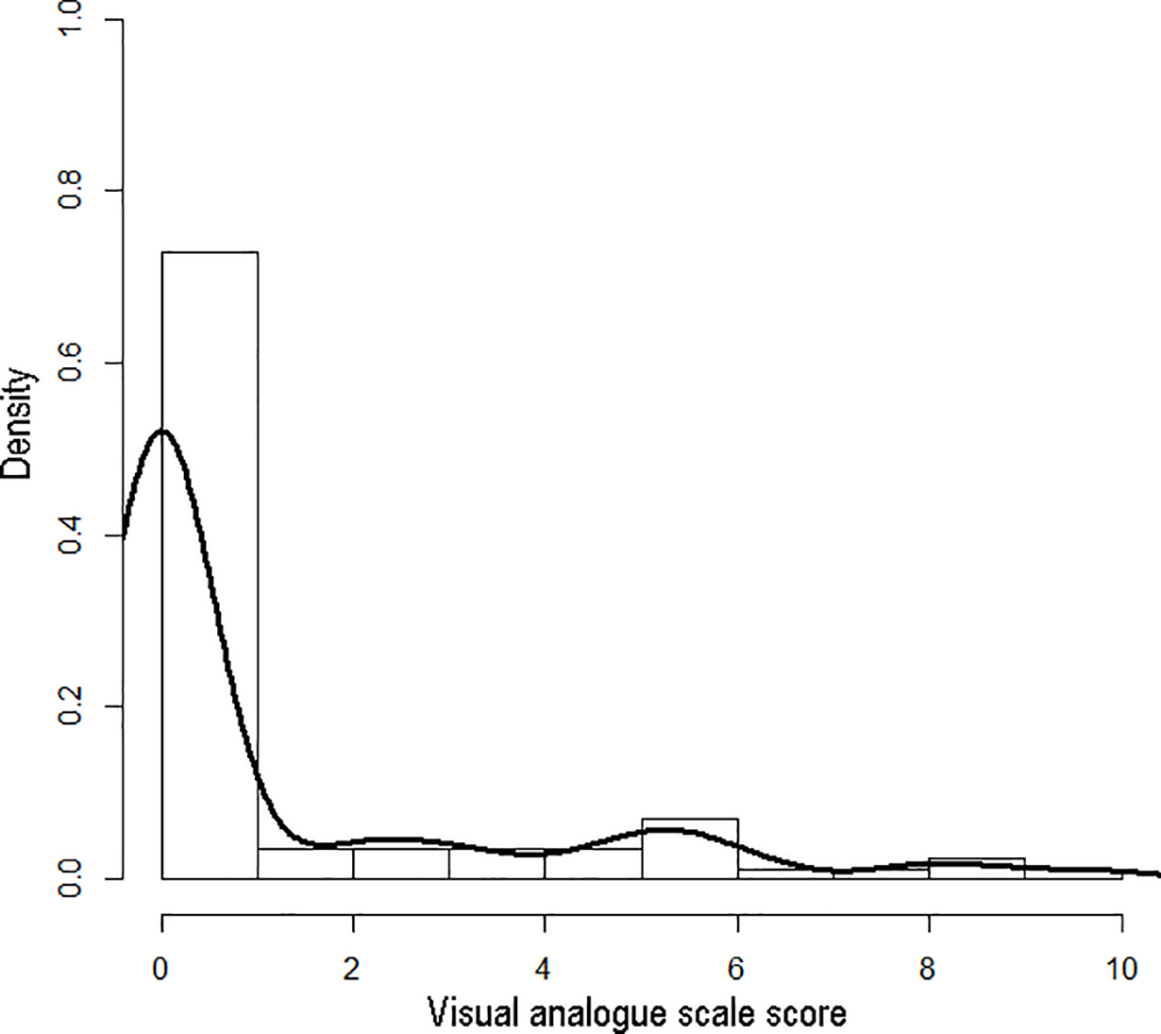
Histogram and density plot of visual analogue scale score of pruritus intensity in the study population (n = 85). The density of vertical axis represents the percentage of study participants.

A total of 85 patients receiving maintenance PD completed this study. During a median follow-up period of 28.0 (1st quartile 27.5, 3rd quartile 28.0) months, 12 patients had technique failure, and 7 died. The baseline characteristics of the participants are summarized in Table 1. The study participants had a mean age of 55.5 years (65% were female) and a median duration of dialysis of 2.3 years. Among the study participants, 24 (28.2%) were classified as having uremic pruritus and had a median VAS score of 4.6. The distribution of the severity of uremic pruritus in the study participants are shown in Fig 1. Comparisons of the clinical and laboratory parameters of participants with uremic pruritus (VAS>0) and without uremic pruritus (VAS = 0) are shown in Table 2. Dietary protein intake was higher and the prevalence of diabetes mellitus was lower among patients with uremic pruritus.

**Table 1:** Demographic and clinical characteristics of the study participants.

Baseline characteristics	Statistics		
Participant number	85		
Sex (female: male)	55 (65%)	:	30 (35%)
Age (year)	55.5	±	12.0
Duration of dialysis (year)	3.1	±	2.7
Etiology of end stage renal disease			
Glomerulonephritis	45 (52.9%)		
Diabetes mellitus	23 (27.1%)		
Systemic lupus erythematosus	5 (5.9%)		
Hypertension	2 (2.4%)		
Interstitial nephritis	2 (2.4%)		
Polycystic kidney disease	2 (2.4%)		
Obstructive uropathy	1 (1.2%)		
Others	5 (5.9%)		

The data are expressed as the mean ± standard deviation or the number (percentage).

**Table 2:** Demographic and clinical characteristics of participants with and without pruritus.

Variable	With pruritus					Without pruritus					*P* value	
Participant number	24					61						
Visual analogue scale score of pruritus intensity	4.6	(	2.5, 5.9	)		0.0	(	0, 0	)		<0.001	*
Age (year)	57.1	±	13.3			54.9	±	11.5			0.46	
Female	18	(	75.0%	)		37	(	60.7%	)		0.21	
Duration of dialysis (year)	2.7	(	0.8, 5.9	)		2.1	(	1.1, 3.7	)		0.63	
Body mass index (kg/m^2^)	22.8	±	4.2			24.2	±	3.3			0.10	
Diabetes mellitus	2	(	8.3%	)		21	(	34.4%	)		0.02	*
Heart failure	1	(	4.2%	)		5	(	8.2%	)		0.51	
Viral hepatitis B	1	(	4.2%	)		7	(	11.5%	)		0.30	
Viral hepatitis C	2	(	8.3%	)		3	(	4.9%	)		0.55	
Daily urine amount (L/day)	0.19	(	0, 0.33	)		0.40	(	0, 0.70	)		0.17	
Weekly total Kt/V	2.11	±	0.27			2.07	±	0.31			0.60	
Weekly renal Kt/V	0.11	(	0, 0.3	)		0.15	(	0, 0.4	)		0.44	
Weekly peritoneal Kt/V	1.91	±	0.30			1.81	±	0.28			0.12	
Total creatinine clearance (L/week/1.73 m^2^)	55.2	(	52.9, 63.5	)		58.0	(	49.9, 68.7	)		0.64	
Renal creatinine clearance (L/week/1.73 m^2^)	6.8	(	0, 23.1	)		8.2	(	0, 23.9	)		0.69	
Peritoneal creatinine clearance (L/week/1.73 m^2^)	49.7	±	7.8			45.9	±	8.5			0.06	
Normalized protein nitrogen appearance (g/kg/day)	1.01	(	0.97, 1.27	)		0.94	(	0.81, 1.13	)		0.05	*
Hematocrit (%)	30.5	±	3.7			30.5	±	4.2			1.00	
Platelets (10^3^/μL)	219	±	72			236	±	69			0.32	
Albumin (g/dL)	3.7	±	0.4			3.7	±	0.5			0.60	
Fasting glucose (mg/dL)	103.5	(	97.0, 118.0	)		100.0	(	90.0, 124.0	)		0.64	
Total cholesterol (mg/dL)	195.7	±	58.7			185.8	±	38.5			0.45	
Triglycerides (mg/dL)	129.0	(	91.0, 227.5	)		113.0	(	74.0, 190.0	)		0.39	
Creatinine (mg/dL)	11.0	±	3.8			11.9	±	3.2			0.25	
Uric acid (mg/dL)	6.7	(	5.5, 7.9	)		6.6	(	6.1, 7.5	)		0.74	
Alanine transaminase (U/L)	15.0	(	10.0, 20.0	)		14.0	(	10.0, 19.0	)		0.83	
Total bilirubin (mg/dL)	0.2	(	0.2, 0.3	)		0.2	(	0.2, 0.3	)		0.64	
Ca × P** (mg/dL × mg/dL)	50.1	±	13.1			50.1	±	12.0			1.00	
Intact parathyroid hormone (pg/mL)	251.2	(	100.8, 792.7	)		264.1	(	122.5, 462.6	)		0.72	
High-sensitivity C-reactive protein (mg/L)	0.3	(	0.1, 0.6	)		0.3	(	0.1, 0.7	)		0.73	

The data are expressed as the mean ± standard deviation for normally distributed continuous variables; as the median (first and third quartiles) for non-normally distributed continuous variables; and as the number (percentage) for categorical variables. *P* values are calculated using the two-sample *t*-test, the Wilcoxon rank-sum test, and the χ^2^ test, respectively.

*P ≤ 0.05.

** Ca × P = Product of albumin-adjusted serum calcium (Ca) and serum phosphorus (P) levels.

### Prognostic importance of uremic pruritus

During follow-up, 19 patients (22.4%) had reached the composite endpoint of PD technique failure or all-cause death. The events among these patients included 6 technique failures and 1 death among those with uremic pruritus (14.8 events per 100 person-years) compared with 6 technique failures and 6 deaths among the patients without pruritus (9.8 events per 100 person-years). The Kaplan-Meier cumulative survival curve in S1 Fig show that compared with nonpruritic patients, those with pruritus showed a trend of lower event-free survival, but the difference did not reach statistical significance (*P* = 0.38).

The results of the multivariable Cox proportional hazards model are shown in Table 3. With the assistance of generalized additive models, we found that a higher VAS score of pruritus intensity was an independent risk factor for technique failure or death (HR, 1.64; 95% CI, 1.18 to 2.28; *P* = 0.003) after adjusting for daily urine amount, peritoneal solute clearance, peritoneal transport, body mass index, heart failure, fasting glucose, platelets, alanine transaminase, creatinine, and uric acid.

**Table 3:** Multivariable Cox proportional hazards model for the composite endpoint of technique failure or all-cause death.

	Hazard	95% Confidence				
Covariate	Ratio	Interval			*P* Value	
Visual analogue scale score of pruritus intensity	1.64	1.18	-	2.28		0.003
Daily urine amount < 0.68 L/day	659.10	30.14	-	14414.90	<	0.001
Peritoneal creatinine clearance < 41.4 L/week/1.73 m^2^	43.70	6.76	-	282.67	<	0.001
4-hour dialysate/plasma creatinine > 0.736	33.12	4.17		262.75	<	0.001
Body mass index ≤ 24.4 kg/m^2^	66.88	6.56	-	681.85	<	0.001
Heart Failure	6.75	1.11	-	40.89		0.038
Fasting glucose (mg/dL)	1.06	1.04	-	1.09	<	0.001
Platelets < 210 10^3^/μL	6.10	1.81	-	20.58		0.004
Alanine transaminase (U/L)	1.07	1.02	-	1.12		0.010
Creatinine < 11.1 mg/dL	6.01	1.18	-	30.54		0.031
Uric acid ≥ 6.3 mg/dL	24.53	3.28	-	184.07		0.002

The Goodness-of-fit assessment, concordance = 0.905, indicates a high discrimination power.

### Determinants of uremic pruritus

**Fig 2:**
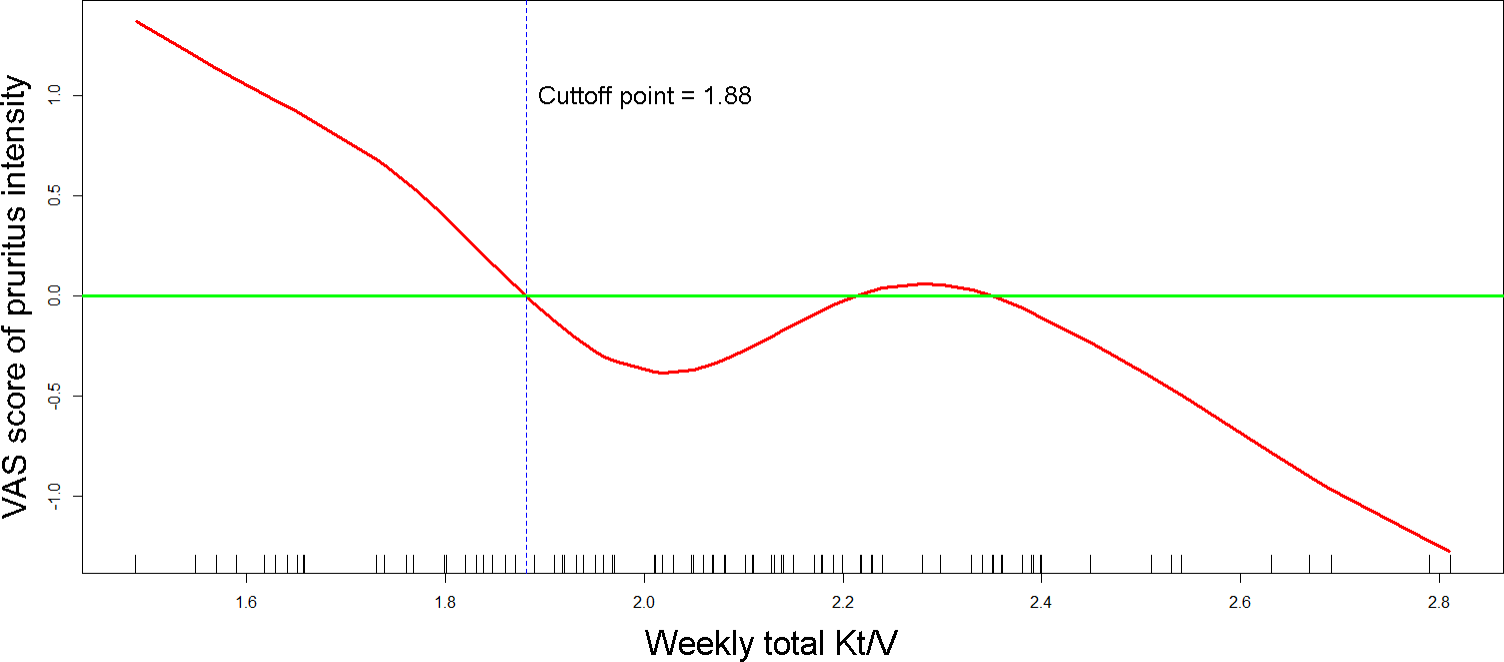
The generalized additive model plot of the relationship between weekly total Kt/V and the visual analogue scale (VAS) score of pruritus intensity. The model of the plot is adjusted for duration of dialysis, normalized protein nitrogen appearance, age, high-sensitivity C-reactive protein, total cholesterol, and intact parathyroid hormone. The little vertical bars on the horizontal axis display the distribution of individual observations. The plot indicates that a weekly total Kt/V < 1.88 is associated with the aggravation of pruritus intensity.

Table 4 shows the results of the multivariable linear regression analyses that were performed to identify the determinants associated with VAS scores of pruritus. The GAMs revealed nonlinear relationships between VAS scores of pruritus intensity and weekly total Kt/V, duration of dialysis, age, high-sensitivity C-reactive protein (hsCRP), and total cholesterol. In Fig 2, the GAM plot reveals that the dose of weekly total Kt/V (in red) intersected the zero VAS score (in green) at a value of 1.88 after adjusted for other covariates. This intersection indicated that a weekly total Kt/V < 1.88 is associated with the aggravation of pruritus intensity. As shown in Table 4, a total Kt/V of less than 1.88, a duration of dialysis longer than 4.1 years, a hsCRP level of > 0.16 mg/L, higher normalized protein nitrogen appearance (nPNA) levels, and higher intact parathyroid hormone (iPTH) levels were independent determinants of higher VAS scores of pruritus intensity. In addition, a patient age between 41.3 and 64.9 years and a cholesterol level between 149.2 and 226.1 mg/dL were associated with lower VAS scores of uremic pruritus.

**Table 4:** Multivariable linear regression analysis of the determinants of visual analogue scale scores of pruritus intensity.

	Parameter	Standard		
Covariate	Estimate	Error	*P* value	
Intercept	-5.530	1.322	<	0.001
Weekly total Kt/V < 1.88	1.165	0.577		0.047
Duration of dialysis > 4.1 (year)	1.153	0.529		0.032
Normalized protein nitrogen appearance (g/kg/day)	3.415	0.989		0.001
41.3 ≤ age < 64.9 (years)	-1.371	0.494		0.007
High-sensitivity C-reactive protein > 0.16 (mg/L)	1.024	0.468		0.032
149.2 ≤ total cholesterol < 226.1 (mg/dL)	-1.743	0.451	<	0.001
Intact parathyroid hormone (pg/mL)	0.002	0.001		0.002

Goodness-of-fit assessment: *R*^*2*^ = 0.377. The *R*^*2*^ value indicates that the correlation between the observed and predicted visual analogue scale scores is 0.614.

## Discussion

In this prospective cohort study assessing the role of uremic pruritus in patients receiving maintenance PD, we found that patients with a higher intensity of pruritus were significantly associated with more technique failure and worse patient survival. In addition, we found that a weekly total Kt/V of less than 1.88, a longer duration of dialysis, a higher dietary protein intake, and higher blood levels of iPTH and hsCRP were independent determinants of higher VAS scores of pruritus intensity in PD patients.

While evidence has shown an association between uremic pruritus and poor outcomes in HD patients, this is the first study to report the detrimental role of uremic pruritus on technique failure and death in PD patients. In chronic HD patients, Narita *et al*. showed that severe pruritus (VAS > 7.0) was independently associated with death (HR 1.60, 95% CI 1.16–2.38) after adjusting for diabetes, age, β2-microglobulin, and albumin [9]. Chen *et al*. also reported that HD patients with moderate/severe uremic pruritus (VAS > 4.0) suffered from higher mortality (HR 3.13, 95% CI 1.60–6.12) after adjusting for age, diabetes, albumin, hemoglobin, phosphate, iPTH, and hsCRP [17]. Among dialysis patients, the unfavorable effects of uremic pruritus may be related to inflammation, which is associated with higher cardiovascular morbidities and mortality [30,31]. The association found between uremic pruritus and hsCRP in our study also supports this hypothesis. Furthermore, uncontrollable itching due to pruritus results in poor skin health and scratch wounds, which may predispose PD patients to higher risks of exit site infection, peritonitis, and technique failure.

In this study, we also noted a higher intensity of uremic pruritus among patients with a weekly total Kt/V < 1.88 compared with those with a higher solute clearance. According to clinical guidelines, the target dose of weekly total Kt/V should be 1.7 or greater to ensure adequate solute clearance in PD [22,23]. This dialysis target is based on randomized clinical trials evaluating the outcomes of mortality or technique failure but not considering pruritus or issues related to quality of life [32,33]. Our previous study suggested a Kt/V value ≥ 1.5 to reduce the intensity of uremic pruritus in HD patients [34], while guidelines on HD suggest a target Kt/V value ≥ 1.4 to reduce mortality [35]. Similarly, the cutoff value of 1.88 for weekly total Kt/V suggested by our study is slightly above the target value of 1.7 that is recommended by PD guidelines, indicating that a solute clearance higher than the current standard may not further improve survival but is able to remove more pruritogenic substances and improve quality of life. Nevertheless, further randomized clinical trials or large cohort studies are necessary for further clarification of the dialyzable pruritogenic substance and the optimal dialysis target for reducing uremic pruritus in PD patients.

In the present study, we used VAS score to measure intensity of uremic pruritus in patients with maintenance PD. To date, no perfect method of objectively measuring pruritus is yet available. As pruritus is a subjective symptom and the VAS score of pruritus intensity is monodimensional, the measured scores are vulnerable to various factors such as emotional change or care satisfaction [36]. However, measuring the VAS score remains a simple and rapid method for assessing pruritus intensity in clinical studies and is therefore widely used.

This study is subject to several limitations. First, as VAS score was measured on a single occasion at baseline, changes of VAS score and time-dependent covariates were not considered in the study. Nevertheless, the VAS score at baseline could serve as one of the useful risk factors for assessing technique failure or death, which reproduce the typical situation of clinical practice. Second, unmeasured confounders are unavoidable due to the observational nature of this cohort study. However, the influence of residual confounders should be small because the concordance in the Cox proportional hazards model and *R*^*2*^ values in the linear regression models were reasonably high. Third, the sample size was relatively small, and the follow-up duration was limited in our study. To increase the number of events and achieve adequate statistical power in survival analysis, we therefore analyzed the composite endpoint. Technique failure and death are related outcomes which both indicate the termination of PD in clinical practice, and have been analyzed as composite events in previous studies[16]. Finally, because the participants were enrolled from a single medical center in Taiwan, the generalizability of the study results to other countries or races may be limited. To clarify the optimal dialysis target considering both survival and quality of life, multicenter randomized clinical trials with a larger sample size and a longer follow-up time, as well as repeated measurement of VAS scores and PD parameters, are required in future studies.

In conclusion, our results show that uremic pruritus is an independent risk factor of technique failure and death in patients receiving maintenance PD. We also found that a weekly total Kt/V < 1.88 is associated with higher intensity of uremic pruritus in PD patients. Randomized clinical trials or larger cohort studies are needed to clarify the optimal dialysis target considering both survival and quality of life in PD patients.

## Supporting information

S1 Fig Kaplan-Meier cumulative survival curves for the composite endpoint of technique failure or all-cause death. Patients were categorized as those with pruritus (VAS score > 0) or those without pruritus (VAS score = 0). VAS, visual analogue scale.
(TIF)
