# Supplementary figures and images for: Prognostic importance and determinants of uremic pruritus in patients receiving peritoneal dialysis: A prospective cohort study
Source: PLoS One. 2018 Sep 5;13(9):e0203474. doi: 10.1371/journal.pone.0203474 (PMC6124771; doi:10.1371/journal.pone.0203474)

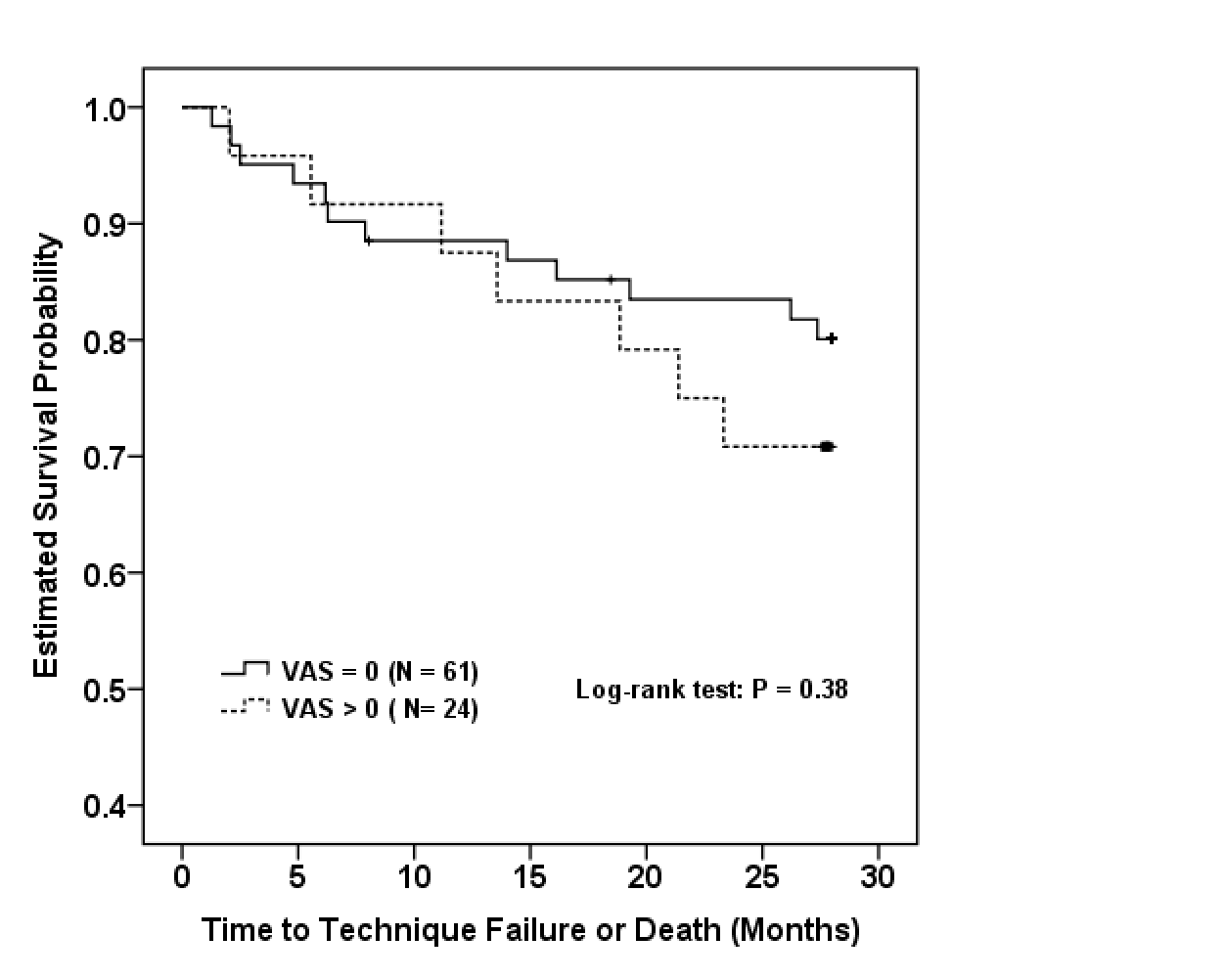

Supplement: S1 Fig — Patients were categorized as those with pruritus (VAS score > 0) or those without pruritus (VAS score = 0). VAS, visual analogue scale. (TIF) [file pone.0203474.s001.tif]
